# A sustainable fertilization strategy to boost maize yield and photosynthetic resilience in saline soils

**DOI:** 10.3389/fpls.2025.1587533

**Published:** 2025-08-08

**Authors:** Hui Zhou, Jiawei Guo, Yongqiang Wang, Jian Wang, Hu Liu

**Affiliations:** ^1^ Yinshanbeilu Grassland Eco-Hydrology National Observation and Research Station, China Institute of Water Resources and Hydropower Research, Beijing, China; ^2^ Institute of Water Resources for Pastoral Area Ministry of Water Resources, Hohhot, China; ^3^ College of Agronomy, Inner Mongolia Agricultural University, Hohhot, China

**Keywords:** organic and inorganic fertilizers, soil, salinization, photosynthesis, antioxidant enzymes, corn

## Abstract

**Introduction:**

Soil salinization is a major constraint to crop production in arid and semi-arid regions. Combined application of organic and inorganic nitrogen (OIN) has been recognized as an effective strategy to improve productivity in saline soils. However, mechanisms underlying yield improvement related to photosynthesis and antioxidant responses remain unclear.

**Methods:**

A field experiment was conducted from 2018 to 2020 in the Hetao Irrigation District of Inner Mongolia using mildly saline soil (S1, EC = 0.68 dS m⁻¹) and moderately saline soil (S2, EC = 1.25 dS m⁻¹). Six nitrogen treatments were applied: no nitrogen (CK), inorganic nitrogen only (U1), and organic nitrogen replacing 25%, 50%, 75%, and 100% of inorganic nitrogen (U3O1, U1O1, U1O3, and O1). Maize growth, photosynthetic parameters, and antioxidant enzyme activities were measured.

**Results:**

Under S1 conditions, OIN significantly enhanced leaf area index (LAI), photosynthetic performance, superoxide dismutase (SOD) activity, and yield, with U1O1 yielding the highest production. Under S2 conditions, growth and photosynthesis were reduced, while malondialdehyde (MDA) content and antioxidant enzyme activities increased. OIN application improved growth, photosynthesis, and catalase (CAT) activity, with O1 achieving the highest yield. Structural equation modeling indicated that yield improvement in S1 was mainly driven by photosynthetic traits, whereas in S2 it resulted from combined effects of growth, photosynthesis, and CAT activity.

**Discussion:**

Overall, OIN application mitigates salinity stress effects on maize, with U1O1 optimal for mildly saline soil and O1 optimal for moderately saline soil. These findings provide insights into managing nitrogen forms to improve crop productivity in saline environments.

## Graphical Abstract

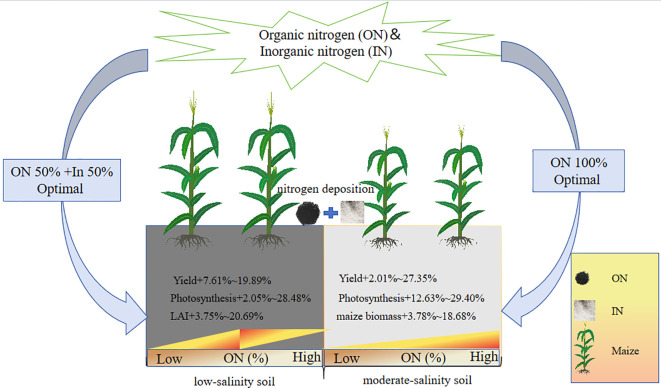

## 1 Introduction

During plant growth, various abiotic and biotic stresses, such as salinity, drought, and pests, frequently disrupt normal physiological processes, thereby affecting plant growth and development (Dixit et al., 2024; El Sabagh et al., 2022; Tripathi et al., 2022; Sattar et al., 2021). Although plants have evolved specific physiological mechanisms to adapt to adverse environmental conditions over time (Nawaz et al., 2023; Alam et al., 2021), their ability to regulate stress responses is limited when the intensity of ecological stress exceeds a certain threshold. This often results in reduced crop yields and, in severe cases, plant mortality. Such effects are particularly pronounced under salt stress (Ahmed et al., 2024; Hamooh et al., 2020; Magdi et al., 2013).

In saline-alkali regions, soil salinity is a major limiting factor for crop growth. Excessive soil salinity deteriorates soil physical properties, severely affects microbial metabolism, and reduces the activity of various soil enzymes, ultimately leading to a decline in soil fertility (Haj-Amor et al., 2022). High salinity also increases the concentration of soil solution, thereby elevating its osmotic potential, which hinders water absorption by plant roots, resulting in physiological drought and, in severe cases, plant mortality (Dos Santos et al., 2022). Current remediation strategies primarily include hydraulic engineering, bioremediation, and chemical amendments to mitigate salt stress and improve soil productivity. While these approaches have advantages, they often suffer from high costs, low efficiency, or secondary pollution. Therefore, identifying suitable materials and technologies to overcome these limitations remains an urgent challenge in managing and remediating saline-alkali soils.

The combined application of organic and inorganic nitrogen (OIN) has gained widespread attention as a sustainable amendment to address various challenges associated with soil salinization (Rezapour et al., 2023; Liu et al., 2023). Previous studies have demonstrated that OIN application is crucial in mitigating non-point source pollution, regulating ecological balance, enhancing soil fertility, and preventing soil degradation (Shahane and Shivay, 2021; Gelaye, 2023; Peng et al., 2023). Moreover, OIN application has been shown to significantly reduce soil salinity and pH while increasing soil organic matter content (Wang et al., 2024; Yu et al., 2024). However, some studies suggest that organic fertilizer application may adversely affect crops in saline soils (Jaime et al., 2016), primarily due to an improper organic-to-inorganic fertilizer ratio. Therefore, determining an optimal OIN ratio tailored to the complex saline soil environment is a key strategy for promoting crop growth and development. Guo et al. (2016) conducted a five-year study in Shandong and found that replacing 25% of chemical fertilizer with organic fertilizer could maintain crop yield. Similarly, Du et al. (2017) reported that in coastal saline-alkali areas, the addition of organic fertilizer to locally optimized fertilization practices enhanced soil microbial activity and enzyme activity, effectively alleviating salt stress and promoting cotton growth, with seed yield reaching or even exceeding that of conventional fertilization. Zhang et al. (2022) studied severely saline-alkali soil in Northeast China. They found that applying organic fertilizer in combination with chemical fertilizer reduced soil salt ion concentrations, improved soil nutrient levels, and increased rice yield. These studies demonstrated that the combined application of OIN contributes to stable or increased crop yields (Guo et al., 2016; Du et al., 2017; Zhang et al., 2022). However, most of the underlying mechanisms have been explored primarily from the perspectives of the rhizosphere environment and crop growth and development. Since photosynthesis directly determines yield, improving photosynthetic performance is a key approach to increasing crop production. In saline-alkali environments, salt-induced osmotic stress reduces stomatal conductance in maize leaves. At the same time, salt stress also accelerates the degradation of enzyme proteins and chlorophyll in the photosynthetic system (Chaves et al., 2009), thereby impairing photosynthetic capacity (Munns and Tester, 2008). However, research on how OIN application affects crop photosynthesis in saline soils remains limited (Roosda et al., 2021; Chen et al., 2022). Additionally, salt stress can induce oxidative stress, inhibiting the ability of antioxidant enzymes to convert superoxide radicals (O_2_^−^) and hydrogen peroxide (H_2_O_2_) into H_2_O and O_2_, which ultimately damages plant cells and leads to yield reduction (Munns and Tester, 2008). Under salt stress conditions, it remains largely unexplored whether OIN application can further enhance the activity of antioxidant enzymes within the antioxidant defense system to mitigate the damage caused by excessive reactive oxygen species (ROS) accumulation on cellular membranes (Wang et al., 2022).

The Hetao Irrigation District in Inner Mongolia is a typical representative of saline-alkali regions in China. Due to natural factors and human activities, secondary salinization of local soils has been intensifying, severely limiting plant photosynthetic capacity and antioxidant defense mechanisms, thereby significantly reducing vegetation cover (Hu et al., 2023; Yang et al., 2024). A well-designed OIN application strategy may provide a feasible, effective, and highly sustainable solution to enhance plant photosynthetic performance, alleviate oxidative stress, and restore the productivity of saline-alkali soils.

Therefore, to elucidate how OIN application affects maize growth parameters, photosynthetic characteristics, malondialdehyde (MDA) content, and antioxidant properties in saline soils, this study conducted a field experiment in maize farmlands with mild to moderate salinization in the Hetao Irrigation District. Five different OIN application ratios were evaluated. A three-year field experiment was conducted to address the following knowledge gap: the underlying mechanisms by which the combined application of OIN influences the coordination between photosynthetic performance, antioxidant defense systems, and yield under different levels of salinity stress remain inadequately understood. The specific objectives of this study were: (1) to investigate the effects of OIN application on photosynthetic efficiency and antioxidant capacity in maize; and (2) to determine the optimal OIN application ratio for achieving high maize yield under varying degrees of soil salinity.

## 2 Materials and methods

### 2.1 Experimental site

The experimental site is located in the Jiefangzha Irrigation Area of the Hetao Irrigation District (40°54′40″N, 107°9′57″E). The soil type is sulfate-chloride saline soil, and the 0–100 cm soil layer consists of silt loam. Mildly saline soil (S1, with an electrical conductivity of 0.68 dS m^-1^) and moderately saline soil (S2, with an electrical conductivity of 1.25 dS m^-1^); the chemical properties of the soils are shown in **Table 1**.

**Table 1:** Basic properties of the tested soils.

Soil	Soil layer	Organic Matter (g kg^−1^)	Total N (g kg^−1^)	Alkaline Hydrolysis N (mg kg^−1^)	Available P (mg kg^−1^)	Available K (mg kg^−1^)	EC (dS m^-1)^	pH
S1	0–20 cm	14.04	1.43	54.68	37.78	199.67	0.68	8.2
	20–40 cm	5.25	0.36	10.25	6.52	102.25	0.60	8.0
	40–60 cm	1.52	0.15	8.15	8.15	30.36	0.59	7.8
	60–100 cm	0.38	0.10	2.53	1.32	10.32	0.51	7.6
S2	0–20 cm	13.05	1.07	46.54	23.58	176.33	1.25	8.3
	20–40 cm	3.15	0.30	11.02	6.33	99.65	1.12	8.1
	40–60 cm	0.87	0.11	6.92	5.95	25.36	0.95	8.0
	60–100 cm	0.26	0.09	2.15	1.15	9.25	0.88	7.8

**Figure 1:**
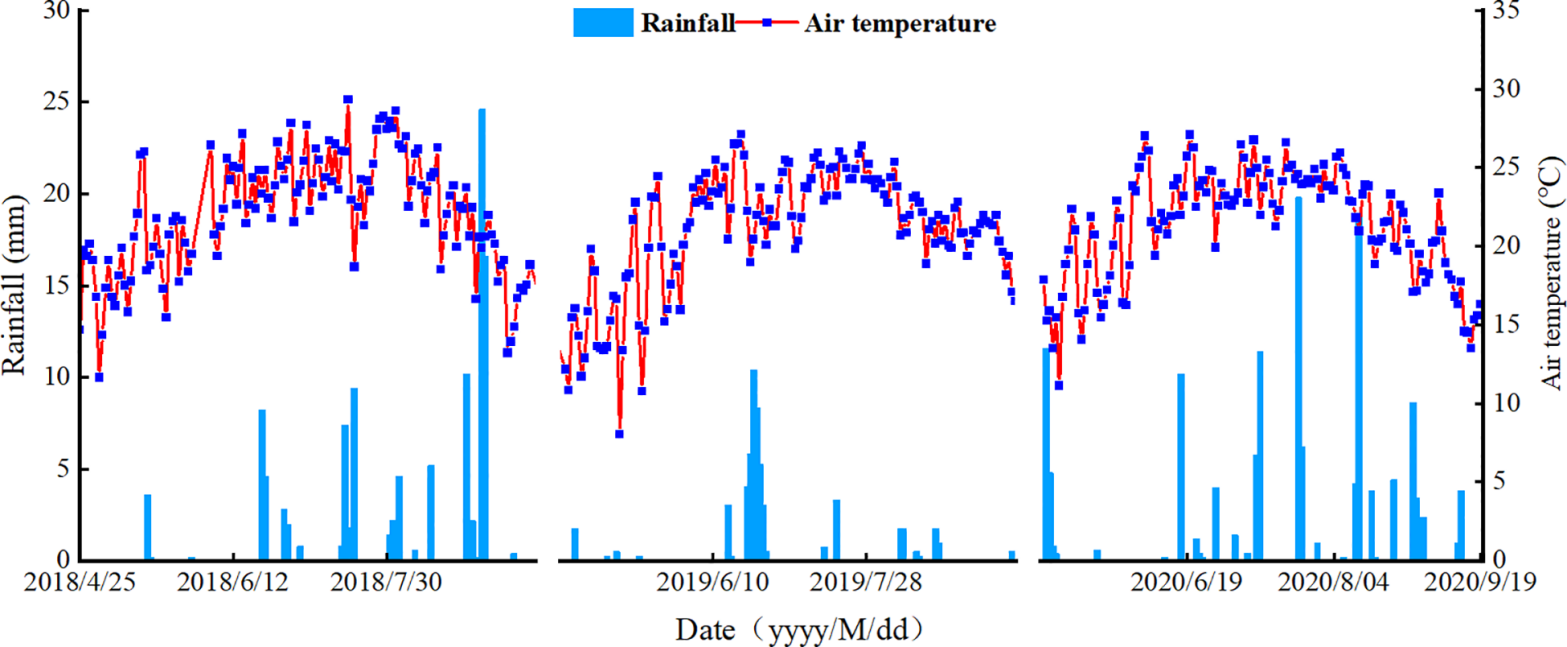
Temperature and rainfall during the 2018–2020 growing seasons.

The soil used in the experiment was collected from the 0–20 cm soil layer after removing surface debris, air-dried, and passed through a 2 mm sieve for subsequent analysis. During the maize-growing seasons from 2018 to 2020, the total precipitation was 111.00 mm, 54.97 mm, and 131.20 mm, respectively. The daily average temperature and precipitation patterns are shown in **Figure 1**.

### 2.2 Experimental design

The experiment was conducted from 2018 to 2020, with maize (*Zea mays* L., variety Neidan 314) as the test crop. The sowing dates were April 27, April 25, and May 5 for the three years, respectively, while the harvest dates were September 13, September 13, and September 19. The irrigation amount was 750 m³ ha^−¹^, and the total nitrogen application rate was 240 kg ha^−¹^. This study employed a randomized complete block design (RCBD). Six treatments were established, including a control without nitrogen application (CK), sole inorganic nitrogen application (U_1_), and organic nitrogen replacing 25%, 50%, 75%, and 100% of the inorganic nitrogen (U_3_O_1_、U_1_O_1_、U_1_O_3_ and O_1%_, respectively), with three replicates per treatment. Each plot had an area of 30 m² (6 m × 5 m), and buffer zones were set between plots to prevent cross-contamination.

The inorganic nitrogen source was urea (46% N), while the organic fertilizer was derived from maize straw, containing 2.5% N, 1% P_2_O_5_, 1% K_2_O, ≥45% organic matter, ≥17% humic acid, and ≥8% sulfur. Organic fertilizer and phosphorus fertilizer (superphosphate at 50 kg ha^−¹^) were applied as basal fertilizers by uniform broadcasting, followed by rotary tillage to a depth of 20 cm. Urea was used in two equal splits: half before sowing and the other half during the jointing stage in conjunction with irrigation.

### 2.3 Measurements and calculations

The grain-filling stage is a critical period for determining maize yield. Therefore, the following indicators were measured on August 5, 2018, August 2, 2019, and August 7, 2020, respectively. The collected leaves were immediately snap-frozen in liquid nitrogen and then quickly transferred to a -80°C freezer for subsequent analysis. The average values over the three years were used for each measured parameter.

#### 2.3.1 Plant height and leaf area index

Five maize plants with uniform growth were selected for measurement in each plot. A meter with an accuracy of 1 mm was used to determine plant height and the length (L, from collar to tip) and maximum width (W) of all green leaves (Kgasago, 2006). The area of a single leaf (ASA) and LAI were calculated using Equations 1, 2, respectively.


(1) ASA=L×W×0.70

(2) $$LAI=\frac{TA\times PD}{1000}$$

Where 0.7 is the regression coefficient of the leaf area as a function of the leaf shape. TA is the total area of a single maize plant in square meters, and PD is the planting density (in plants per hectare).

#### 2.3.2 Aboveground biomass

Five maize plants were selected from each plot. The collected aboveground biomass was first inactivated at 105°C for 30 minutes in a ventilated drying oven, followed by drying at 75°C until a constant weight was reached. The dry weight per plant was then determined and expressed in g/plant (Liao et al., 2022).

#### 2.3.3 Chlorophyll content

Five maize plants were selected from each plot, and the relative chlorophyll content (SPAD value) was measured using a SPAD-502 chlorophyll meter. The measurement position was consistently set at the middle of the ear leaf (Kang et al., 2005).

#### 2.3.4 Grain yield

During maize harvest, 10 plants were selected from each plot for air drying and threshing. Grain yield was measured and adjusted to a standard grain moisture content of 14% (Xu et al., 2022).

#### 2.3.5 Photosynthetic performance

Leaf net photosynthetic rate, stomatal conductance, intercellular CO_2_ concentration, and transpiration rate were measured using a LI-6400XT photosynthesis system. During clear weather conditions, measurements were conducted on the ear leaf between 9:30 and 11:30 AM. The leaf chamber was illuminated with a red-blue light source, and the photosynthetically active radiation (PAR) was set to 1000 μmol·m−²·s^−¹^, without artificial control of CO_2_ concentration during measurement (Merritt, 2019).

#### 2.3.6 Antioxidant system indicators

Lipid peroxidation was assessed by measuring the absorbance of fresh leaf samples (1.0 g) at 532 nm and 600 nm, and expressed as malondialdehyde (MDA) content (Du and Bramlage, 1992). Peroxidase (POD) and catalase (CAT) activities were determined using the guaiacol colorimetric method (Luo et al., 2013), with one unit (U) of enzyme activity defined as a 0.10 change in absorbance at 470 nm per minute. Superoxide dismutase (SOD) activity was measured by the nitroblue tetrazolium (NBT) method (Beyer and Fridovich, 1987), recording absorbance at 560 nm. One unit (U) of SOD activity was defined as the amount of enzyme required to inhibit 50% of the photochemical reduction of NBT.

### 2.4 Data processing and analysis

Data were organized and processed using Excel 2016. MDS and PCA selection and correlation analysis of crop indicators were performed using SPSS 20.0 (IBM Corp., Armonk, NY, USA). Multiple comparisons (Least Significant Difference) were conducted for the data. Graphs were created using Origin (ver. 9.5; OriginLab Corp., Northampton, MA, USA). Structural equation modeling (SEM) was performed using the ‘lavaan’ package in R (version 4.0.3) to determine the comprehensive effects (both direct and indirect) of soil microbial biomass pools and phosphorus (P) fractions on P uptake. The model fit was evaluated using the maximum likelihood (χ²) goodness-of-fit test, p-values, and the root mean square error of approximation (RMSEA).

## 3 Results and analysis

### 3.1 Growth parameters

**Figure 2:**
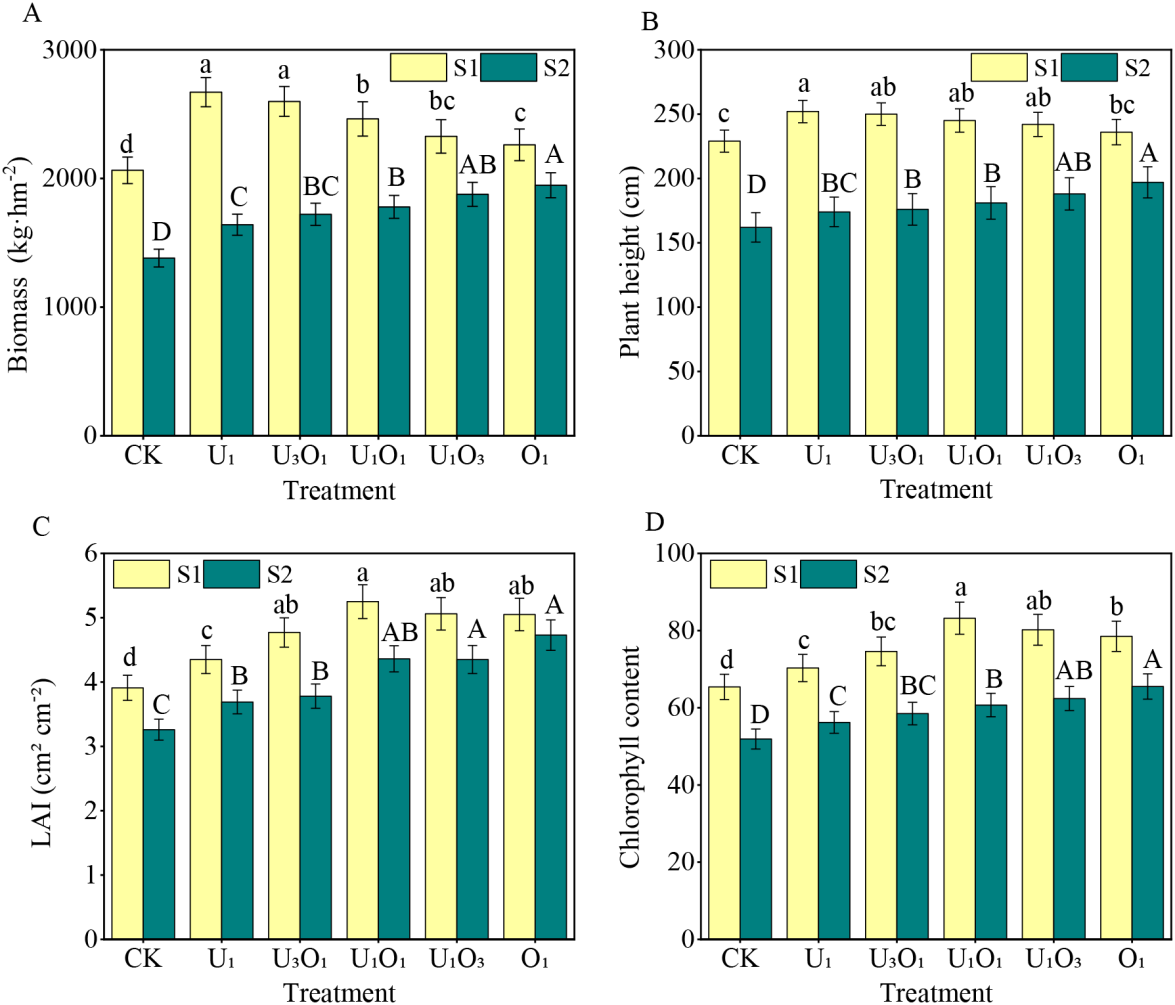
Changes in maize biomass **(A)**, plant height **(B)**, leaf area index **(C)**, and chlorophyll content **(D)** under different fertilization treatments. Different capital and lowercase letters respectively indicated significant difference between different treatments of mild and moderate salinity soil (P<0.05). CK: no nitrogen applied (control); U_1_: sole application of inorganic nitrogen; U_3_O_1_: 25% of the inorganic nitrogen replaced by organic nitrogen; U_1_O_1_: 50% of the inorganic nitrogen replaced by organic nitrogen; U_1_O_3_: 75% of the inorganic nitrogen replaced by organic nitrogen; O_1_: 100% of the inorganic nitrogen replaced by organic nitrogen.

As shown in **Figure 2**, increased salinity significantly reduced maize growth parameters. Compared with S1 soil, maize biomass, plant height, leaf area index, and chlorophyll content in S2 soil decreased by 13.87%–38.56%, 16.53%–30.95%, 6.34%–22.67%, and 16.56%–27.04%, respectively.

Under S1 soil conditions, nitrogen application significantly improved maize growth indicators. Compared with CK, nitrogen treatments increased maize biomass, plant height, leaf area index, and chlorophyll content by 9.57%–29.42%, 3.06%–10.04%, 11.25%–34.27%, and 7.49%–27.22%, respectively. Among them, the U_1_ treatment resulted in the highest biomass and plant height, which significantly exceeded those of the O_1_ treatment by 18.11% and 6.78%, respectively (P< 0.05), and showed a decreasing trend with increasing proportion of organic nitrogen. The highest leaf area index and chlorophyll content were observed under the U_1_O_1_ treatment, which were 3.75%–20.69% and 3.74%–18.35% higher than other nitrogen treatments, respectively (P< 0.05).

Under S2 soil conditions, nitrogen treatments also significantly enhanced maize biomass, plant height, leaf area index, and chlorophyll content, with increases of 18.83%–41.02%, 7.41%–21.60%, 13.19%–45.09%, and 8.29%–26.20%, respectively. Moreover, under S2 soil, maize growth parameters increased with the proportion of organic nitrogen. The O_1_ treatment resulted in the highest biomass, plant height, leaf area index, and chlorophyll content, exceeding other nitrogen treatments by 3.78%–18.68%, 4.78%–13.22%, 8.74%–28.18%, and 4.97%–16.55%, respectively.

### 3.2 Photosynthetic characteristics of maize

**Figure 3:**
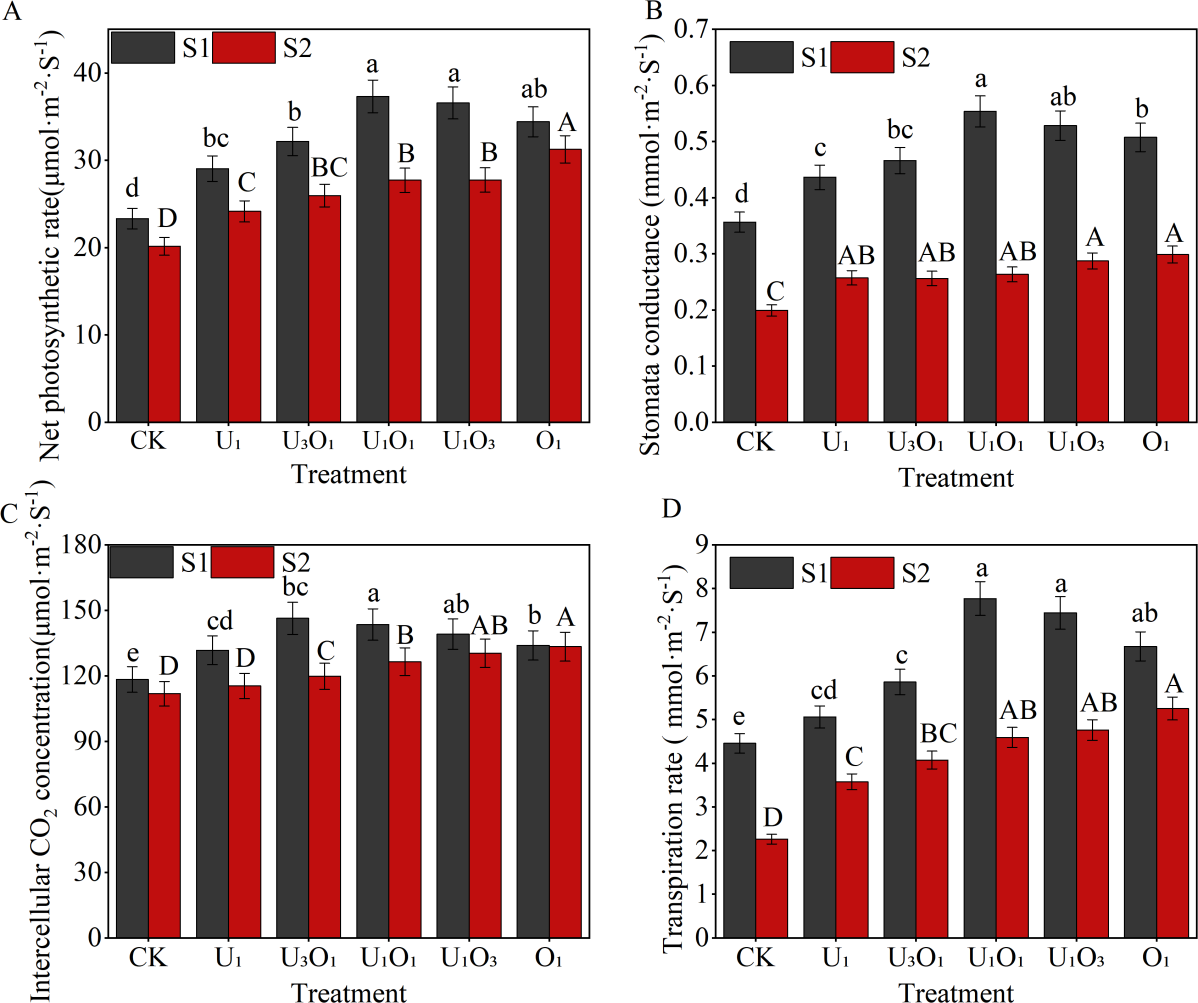
Changes in maize net photosynthetic rate **(A)**, stomatal conductance **(B)**, intercellular CO_2_ concentration **(C)**, and transpiration rate **(D)** under different fertilization treatments. Different capital and lowercase letters respectively indicated significant difference between different treatments of mild and moderate salinity soil (P<0.05). CK: no nitrogen applied (control); U_1_: sole application of inorganic nitrogen; U_3_O_1_: 25% of the inorganic nitrogen replaced by organic nitrogen; U_1_O_1_: 50% of the inorganic nitrogen replaced by organic nitrogen; U_1_O_3_: 75% of the inorganic nitrogen replaced by organic nitrogen; O_1_: 100% of the inorganic nitrogen replaced by organic nitrogen.

As shown in **Figure 3**, increased salinity significantly inhibited maize photosynthesis. Compared with S1 soil, the net photosynthetic rate, stomatal conductance, intercellular CO_2_ concentration, and transpiration rate in S2 soil decreased by 9.16%–25.70%, 41.00%–52.40%, 0.44%–18.13%, and 21.29%–49.21%, respectively, across all treatments.

The combined application of organic and inorganic nitrogen fertilizers had a positive effect on photosynthetic parameters in both S1 and S2 soils. Under S1 soil conditions, all fertilization treatments significantly improved the net photosynthetic rate, stomatal conductance, intercellular CO_2_ concentration, and transpiration rate, with increases of 24.52%–59.99%, 22.39%–55.32%, 11.30%–23.64%, and 13.53%–74.33%, respectively, compared with CK. Among them, the U_1_O_1_ treatment achieved the highest values for net photosynthetic rate, stomatal conductance, and transpiration rate, exceeding other nitrogen treatments by 2.05%–28.48%, 4.80%–26.91%, and 4.40%–53.56%, respectively, with no significant difference from the U_1_O_3_ treatment (P< 0.05).

Under S2 soil conditions, fertilization treatments also significantly enhanced photosynthetic performance. Compared with CK, the net photosynthetic rate, stomatal conductance, intercellular CO_2_ concentration, and transpiration rate increased by 19.84%–55.07%, 29.25%–50.06%, 3.14%–19.23%, and 57.96%–132.07%, respectively. Moreover, the application of organic nitrogen effectively alleviated the inhibitory effects of salt stress on maize photosynthesis. Among all treatments, O_1_ showed the best performance, with net photosynthetic rate, stomatal conductance, intercellular CO_2_ concentration, and transpiration rate being 12.63%–29.40%, 4.01%–16.56%, 2.29%–15.60%, and 10.37%–46.92% higher, respectively, than those under sole inorganic nitrogen application (P< 0.05).

### 3.3 Malondialdehyde content and antioxidant enzyme activities

**Figure 4:**
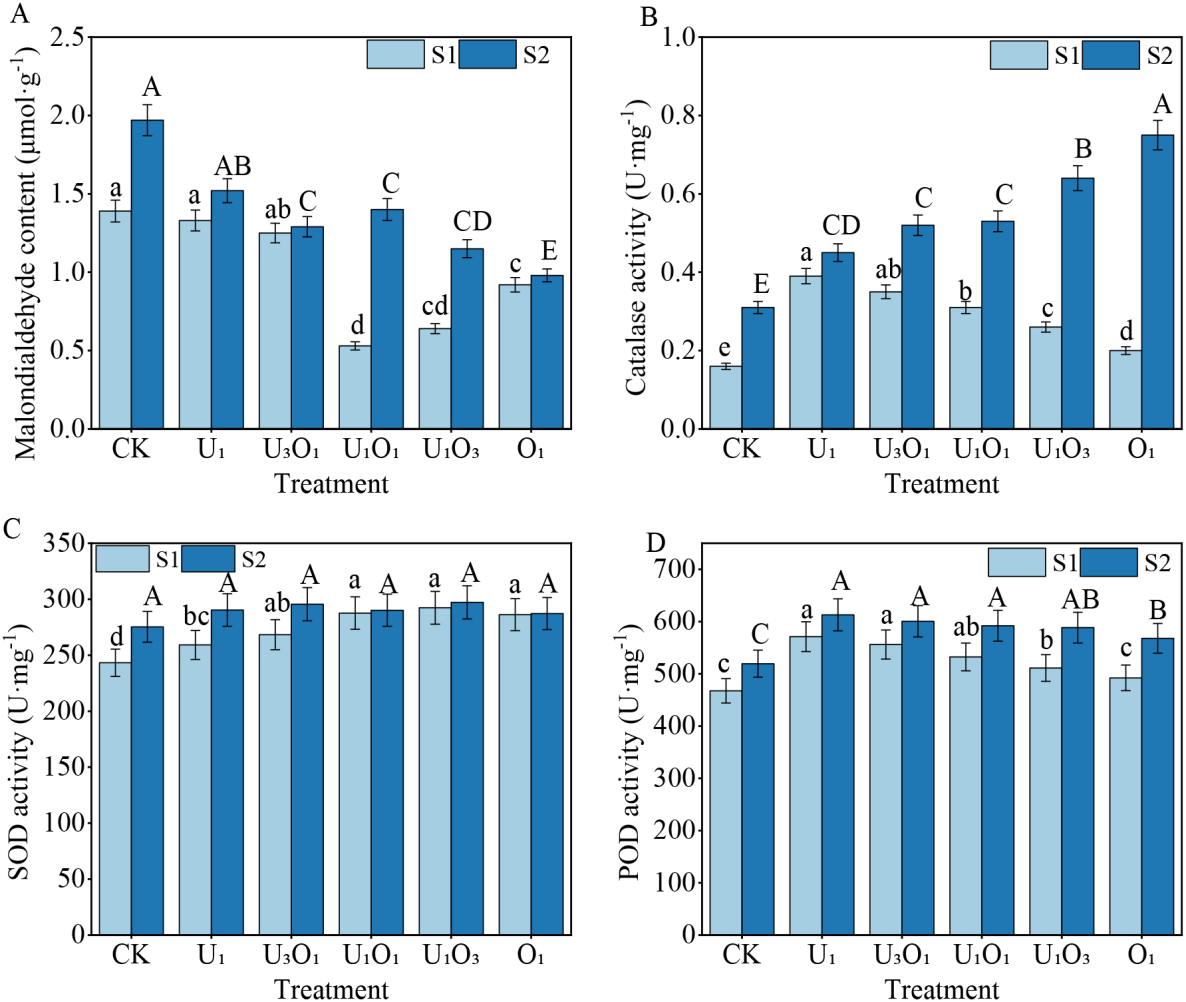
Changes in maize MDA content **(A)**, CAT activity **(B)**, SOD activity **(C)**, and POD activity **(D)** under different fertilization treatments. Different capital and lowercase letters respectively indicated significant difference between different treatments of mild and moderate salinity soil (P<0.05). CK: no nitrogen applied (control); U_1_: sole application of inorganic nitrogen; U_3_O_1_: 25% of the inorganic nitrogen replaced by organic nitrogen; U_1_O_1_: 50% of the inorganic nitrogen replaced by organic nitrogen; U_1_O_3_: 75% of the inorganic nitrogen replaced by organic nitrogen; O_1_: 100% of the inorganic nitrogen replaced by organic nitrogen.

As shown in **Figure 4**, increased salinity induced stress in maize plants. Compared with S1 soil, the malondialdehyde (MDA) content in maize grown in S2 soil increased by 3.20%–164.15%, indicating enhanced oxidative stress. Meanwhile, antioxidant enzyme activities were elevated under salt stress: CAT, SOD, and POD activities in S2 soil were 13.33%–73.33%, 0.34%–11.66%, and 6.82%–13.30% higher, respectively, than those in S1 soil.

Under S1 soil conditions, nitrogen treatments significantly reduced MDA content by 4.32%–61.87% compared with the control (CK), while CAT, SOD, and POD activities increased by 25.00%–143.75%, 6.56%–20.20%, and 5.33%–22.20%, respectively. With increasing organic nitrogen application, MDA content first decreased and then increased, reaching the lowest level under the U_1_O_1_ treatment, which was significantly 60.15% lower than that under the U_1_ treatment (P< 0.05). In contrast, CAT and POD activities showed a decreasing trend with increasing organic nitrogen input, with CAT and POD activities under the U_1_ treatment being 95.00% and 16.01% higher, respectively, than those under the O_1_ treatment (P< 0.05). SOD activity initially increased and then decreased with increasing organic nitrogen ratio; there were no significant differences among the U_1_O_1_, U_1_O_3_, and O_1_ treatments, but all showed significantly higher SOD activity than U_1_ by 10.96%, 12.80%, and 10.42%, respectively (P< 0.05).

Under S2 soil conditions, all nitrogen treatments reduced MDA content by 22.84%–50.25% compared with CK, while CAT, SOD, and POD activities increased by 45.16%–141.94%, 4.30%–7.94%, and 9.33%–18.01%, respectively. The combined application of organic nitrogen effectively alleviated the inhibitory effects of salt stress. MDA content decreased with increasing organic nitrogen input, with the O_1_ treatment showing the lowest value—14.78%–35.53% lower than other fertilization treatments (P< 0.05). CAT activity increased gradually with increasing organic nitrogen rate, peaking in the O_1_ treatment, which was significantly 17.19%–66.67% higher than other treatments (P< 0.05). No significant differences in SOD activity were observed among treatments. POD activity decreased slightly with increasing organic nitrogen input, although differences among treatments were not statistically significant.

### 3.4 Maize yield

**Figure 5:**
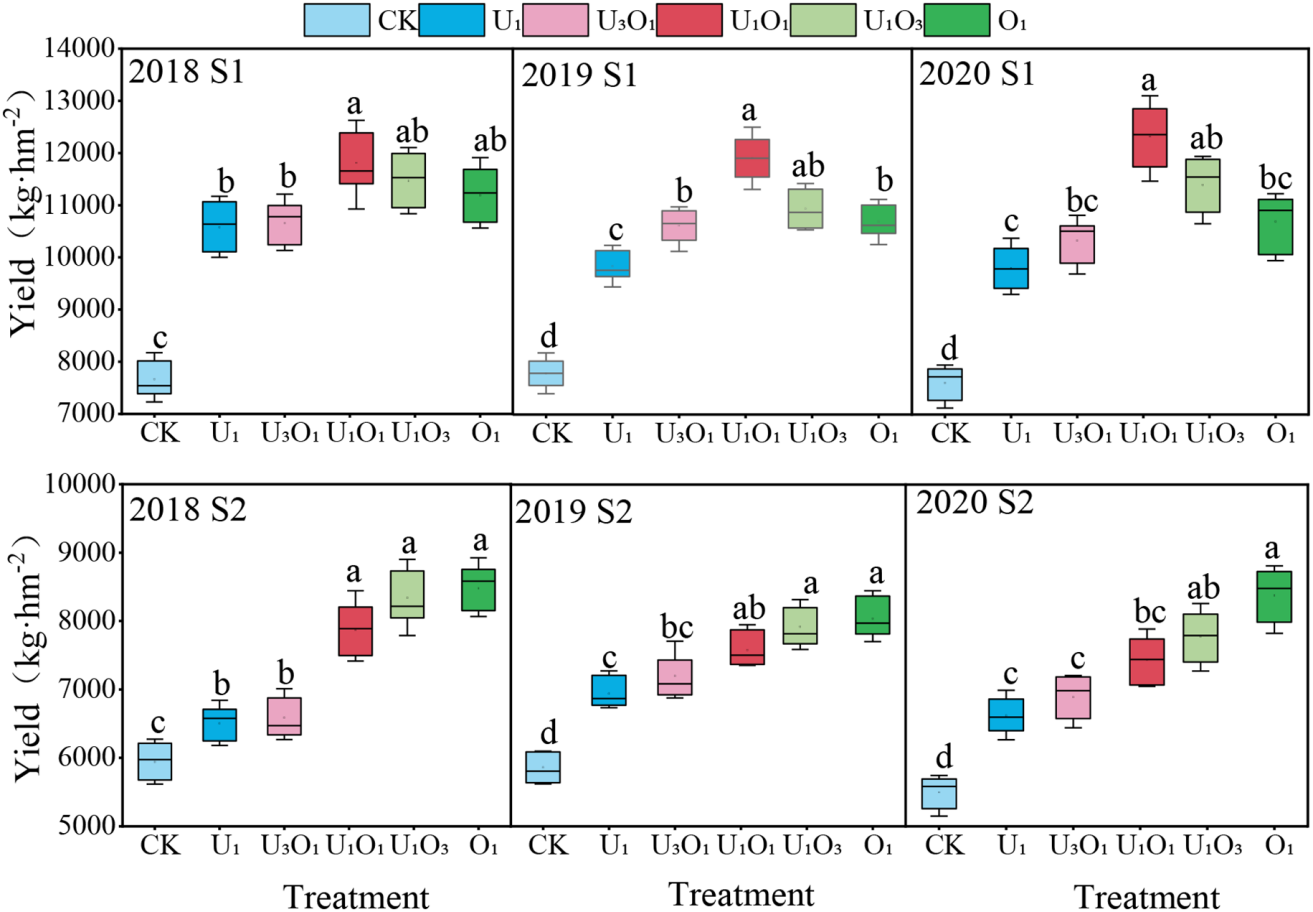
Maize crop yield response to nitrogen application. Different letters indicate significant differences among all treatments at a significance level of P< 0.05. Data represent the means of three replications. CK: no nitrogen applied (control); U_1_: sole application of inorganic nitrogen; U_3_O_1_: 25% of the inorganic nitrogen replaced by organic nitrogen; U_1_O_1_: 50% of the inorganic nitrogen replaced by organic nitrogen; U_1_O_3_: 75% of the inorganic nitrogen replaced by organic nitrogen; O_1_: 100% of the inorganic nitrogen replaced by organic nitrogen.

As shown in **Figure 5**, salt stress significantly inhibited maize yield. Compared with S1 soil, maize yield in S2 soil decreased by 22.76%–36.50% (P< 0.05). Nitrogen application significantly increased maize yield, with increases of 31.19%–57.28% under S1 soil and 18.25%–50.59% under S2 soil compared to CK.

Under S1 soil conditions, maize yield increased initially and then declined with the rising proportion of organic nitrogen, with the highest yield observed under the U_1_O_1_ treatment, which was significantly 7.61%–19.89% higher than the other nitrogen treatments (P< 0.05).

Under S2 soil conditions, the addition of organic nitrogen effectively alleviated the negative effects of salt stress on maize yield. Yield increased with increasing organic nitrogen ratio, and the O_1_ treatment produced the highest yield, which was 2.01%–27.35% higher than other nitrogen treatments.

### 3.5 Correlation analysis

**Figure 6:**
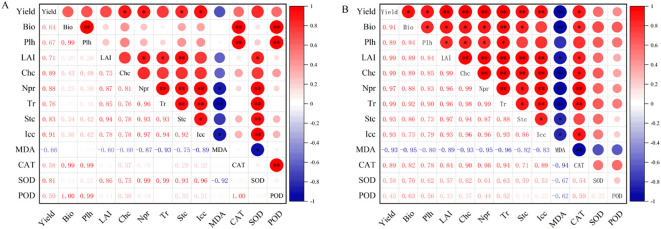
Correlation analysis of maize growth and physiological parameters under **(A)** mild and **(B)** moderate salinity stress. Sample size = 90. indicates significance at P < 0.05; * indicates significance at P < 0.01. Bio: biomass; Plh: plant height; CHc: chlorophyll content; Npr: net photosynthetic rate; Tr: transpiration rate; Stc: stomatal conductance; Icc: intercellular CO_2_ concentration.

Pearson correlation analysis (**Figure 6**) revealed that, in S1 soil, maize yield showed no significant correlation with biomass and plant height (*P* > 0.05), but had a highly significant positive correlation with leaf area index (LAI) and chlorophyll content (*P*< 0.01). Maize yield also exhibited a highly significant positive correlation with all photosynthetic parameters (*P*< 0.01). Maize yield showed a highly significant negative correlation with MDA content (*P*< 0.01) and a significant positive correlation with SOD activity (*P*< 0.05), but no significant correlation with CAT and POD activities (*P* > 0.05). There was a highly significant positive correlation between maize biomass and plant height (*P*< 0.01), but no significant correlation with LAI and chlorophyll content (*P* > 0.05). Maize biomass and plant height had no significant correlation with photosynthetic parameters (*P* > 0.05). In contrast, maize LAI and chlorophyll content showed significant (*P*< 0.05) or highly significant positive correlations (*P*< 0.01) with all photosynthetic parameters. Maize biomass and plant height did not significantly correlate with MDA content (*P* > 0.05). In contrast, maize LAI and chlorophyll content exhibited a highly significant negative correlation with MDA content (*P*< 0.01). All photosynthetic parameters had a highly significant positive correlation (*P*< 0.01). All photosynthetic parameters were highly negatively correlated with MDA content (*P*< 0.01), and highly positively correlated with SOD activity (*P*< 0.01), while showing no significant correlation with CAT and POD activities (*P* > 0.05).

In S2 soil, maize yield exhibited significant (*P*< 0.05) or highly significant positive correlations (*P*< 0.01) with all growth parameters and photosynthetic characteristics. Maize yield had a highly significant negative correlation with MDA content (*P*< 0.01) and a highly significant positive correlation with CAT activity (*P*< 0.01), but no significant correlation with SOD and POD activities (*P* > 0.05). All growth parameters showed highly significant positive correlations (*P*< 0.01). Additionally, all growth parameters had significant (*P*< 0.05) or highly significant positive correlations (*P*< 0.01) with photosynthetic parameters. All growth parameters exhibited a highly significant negative correlation with MDA content (*P*< 0.01), a significant positive correlation with CAT activity (*P*< 0.05), and no significant correlation with SOD and POD activities (*P* > 0.05). All photosynthetic parameters were highly positively correlated (*P*< 0.01), with a highly significant negative correlation with MDA content (*P*< 0.01) and a highly significant positive correlation with CAT activity (*P*< 0.01), while showing no significant correlation with SOD and POD activities (*P* > 0.05).

### 3.6 Pathways affecting maize yield

**Figure 7:**
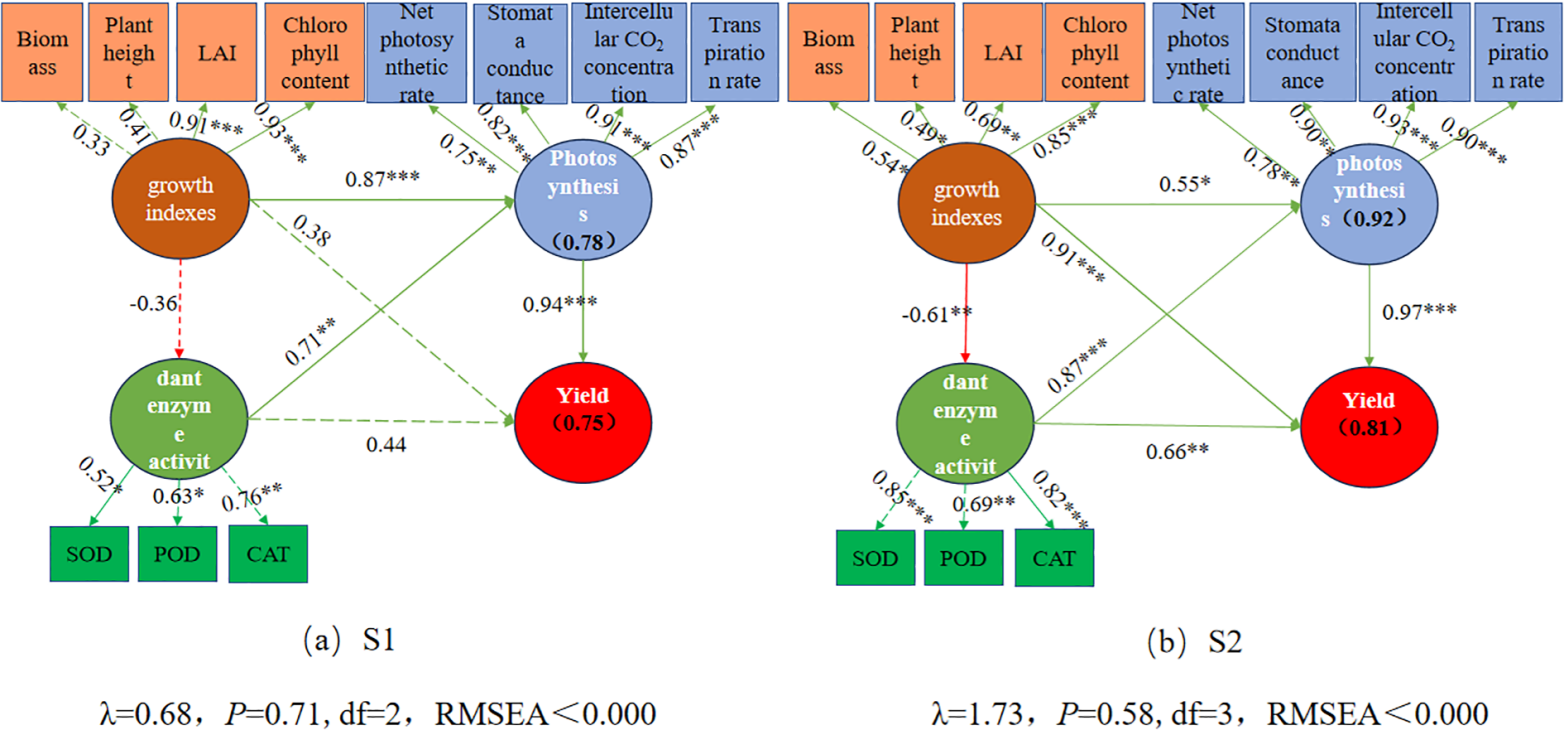
Structural equation model (SEM) showing the direct and indirect effects of growth indexes, Photosynthesis, and antioxidant enzyme activity on maize yield in the S1 and S2 soil. The boxes’ numbers show the variance percentages explained by the predictor variables. The solid and dashed arrows indicate significant and nonsignificant relationships, respectively. The green and red arrows indicate positive and negative relationships, respectively. The numbers above the arrows denote the standardized path coefficients (*p< 0.05, **p< 0.01, ***p< 0.001). χ2, chi-square; p, probability level; df, degrees of freedom.

Structural equation modeling (SEM) revealed that in both S1 and S2 soils, maize growth parameters, photosynthetic parameters, and antioxidant indicators significantly influenced maize yield, either directly or indirectly (**Figure 7**). The final SEM explained 75% and 81% of the total variation in maize yield in S1 and S2 soils, respectively (**Figures 7a, b**). The influence of maize growth and antioxidant indicators on maize yield was more significant in S2 soil than in S1 soil, indicating that maize growth traits and antioxidant characteristics contribute more to maize yield in high-salinity soils. Maize photosynthetic parameters significantly contributed to maize yield in both S1 and S2 soils. Moreover, maize growth parameters positively affected photosynthetic characteristics in both S1 and S2 soils (0.87, 0.55), but hurt antioxidant indicators (0.36, 0.61). These results demonstrate that combining organic and inorganic nitrogen promotes maize yield by enhancing maize growth traits and improving photosynthetic performance. Among multiple variables, the effect of nitrogen application on crop yield in S1 soil is best explained by photosynthetic indicators (**Figure 7a**), whereas in S2 soil, maize growth parameters, photosynthetic indicators, and CAT activity provide the best explanation (**Figure 7b**).

## 4 Discussion

### 4.1 Growth parameters

The external morphology and growth vigor of maize were intuitive indicators of its response to salt stress. This study showed that increased soil salinity significantly inhibited maize growth and development (**Figure 2**). On the one hand, high soil salt concentrations altered the osmotic potential, thereby affecting water and nutrient uptake and suppressing crop growth (Hailu and Mehari, 2021). On the other hand, the soil type in the study area was classified as sulfate–chloride saline soil, in which certain specific ions (e.g., Na^+^ and Cl^−^) at excessive concentrations exerted toxic effects on crops by infiltrating cells and causing protoplasmic aggregation, which in turn led to a reduction in chlorophyll content (Gupta and Dagar, 2016).

This study also found that nitrogen application significantly improved maize growth parameters, with more pronounced effects observed under S2 soil conditions, suggesting that nitrogen input alleviated the adverse impacts of salinity on crop growth (Alkharabsheh et al., 2021). To achieve high yields, the ideal maize growth pattern involved a relatively smaller photosynthetic area during vegetative growth to avoid excessive elongation, and a larger photosynthetic area during the critical period of yield formation (Feng et al., 2024). In this study, as the proportion of organic nitrogen increased, plant height and biomass in S1 soil gradually declined, whereas a proportion of organic nitrogen exceeding 50% significantly enhanced chlorophyll content and leaf area index (**Figure 2**). Correlation analysis showed that maize yield was significantly positively correlated with chlorophyll content and leaf area index (P< 0.01) (**Figure 6**). This indicated that under low-salinity conditions, the combined application of organic fertilizer prevented excessive vegetative growth and maintained a high green leaf area and elevated chlorophyll content during grain filling, thereby prolonging the grain-filling period and promoting yield formation. These results were consistent with those of Zhang et al. (2023), who found that the application of organic fertilizer in combination with reduced NPK rates improved leaf chlorophyll content and dry matter accumulation, and extended the grain-filling duration.

In S2 soil, all maize growth indicators increased with the rising proportion of organic fertilizer (**Figure 2**), indicating that high salinity strongly suppressed crop growth, and thus required greater organic matter input to mitigate osmotic stress induced by salinity. However, Zhao et al. (2023) reported that the sole application of organic fertilizer resulted in low nitrogen mineralization and a prolonged mineralization period, which might have been related to the type of organic material applied and the high salt content of the soil.

### 4.2 Photosynthetic characteristics of maize

Photosynthesis is the physiological foundation for maize growth and yield formation. In saline soils, both salinity and nutrient limitations are the two main factors restricting photosynthetic performance. This study found that salt stress suppressed the net photosynthetic rate, transpiration rate, and stomatal conductance in maize leaves, with trends consistent with changes in intercellular CO_2_ concentration (**Figure 3**). These findings were similar to those reported by Ren et al. (2021) for ryegrass and alfalfa, indicating that the primary cause of salt-induced reductions in photosynthesis was stomatal limitation (Hussain et al., 2021; Zahra et al., 2022). Correlation analysis showed that stomatal conductance was highly significantly and positively correlated with both net photosynthetic rate and transpiration rate (P< 0.01), suggesting that the decline in stomatal conductance further reduced photosynthetic performance, consistent with previous findings (Wei et al., 2010).

In slightly saline soils, intercellular CO_2_ concentration in maize first increased and then decreased with increasing organic nitrogen input. This indicated that under low salinity, moderate organic nitrogen application significantly enhanced net photosynthetic rate, while excessive organic fertilizer might have reduced effective soil porosity (Xu et al., 2021), disrupted plant metabolic processes, and ultimately suppressed certain photosynthetic functions (Liu et al., 2024). Correlation analysis showed that in slightly saline soils, photosynthetic traits of maize were not significantly correlated with plant height or biomass, but were highly significantly and positively correlated with leaf area index, chlorophyll content, and yield (P< 0.01). This further demonstrated that under lower salinity conditions, moderate organic fertilizer application helped maintain a high photosynthetic rate during the reproductive stage, which supported canopy development and contributed to improved yield formation under slightly saline conditions. In moderately saline soils, photosynthetic traits were significantly (P< 0.05) or highly significantly (P< 0.01) positively correlated with growth indicators and yield, suggesting that under higher salt stress, greater organic fertilizer inputs were needed to promote plant growth, enhance photosynthetic performance, and ultimately increase yield.

### 4.3 Malondialdehyde content and antioxidant enzyme activities

In general, an efficient antioxidant system within plants is capable of counteracting damage caused by reactive oxygen species (ROS) and maintaining redox homeostasis in cells, thereby supporting normal photosynthesis and other metabolic processes. However, salt stress increases osmotic pressure in plant tissues, disrupts ionic balance, and damages the structure and function of cell membranes. As a result, the antioxidant defense system may be insufficient to eliminate excessive ROS, leading to oxidative damage (Singh et al., 2024). Malondialdehyde (MDA) is a key indicator of oxidative stress in plants, and its concentration directly reflects the degree of membrane lipid peroxidation. In this study, MDA levels under mild salt stress were significantly lower than those under moderate salt stress (**Figure 4**), indicating that maize suffered more severe stress under higher salinity.

Under stress conditions, plants develop adaptive mechanisms. Among them, superoxide dismutase (SOD), peroxidase (POD), and catalase (CAT) are the major antioxidant enzymes responsible for scavenging ROS. These enzymes work synergistically to convert O_2_^−^ and H_2_O_2_ into H_2_O and O_2_, thereby mitigating oxidative damage to the cell membrane system (Ahmad and Kamal, 2021). Their activity levels can reflect metabolic changes and stress tolerance in plants under adverse conditions. The results of this study showed that under moderate salinity, maize exhibited higher SOD, POD, and CAT activities compared to those in mildly saline soils, suggesting that maize responded actively to salt stress by enhancing antioxidant enzyme activity to alleviate damage. These findings were consistent with those of Zhang et al. (2020).

In mildly saline soils, the addition of organic fertilizer significantly enhanced SOD activity in maize. Since elevated SOD activity inevitably leads to increased H_2_O_2_ production, it was notable that CAT and POD activities—enzymes responsible for H_2_O_2_ elimination—generally declined with increasing organic fertilizer input. However, photosynthetic performance under these conditions was better than that observed with sole inorganic nitrogen application. This may be because under low-salinity conditions, the toxicity of free radicals had a stronger impact on maize than H_2_O_2_, and the addition of organic fertilizer promoted SOD activity to eliminate excess radicals. Similar findings regarding the influence of organic fertilizers on antioxidant enzyme activity were also reported by Zhang et al. (2025). Under moderate salinity, the addition of organic fertilizer did not significantly alter SOD activity, but CAT activity increased significantly while POD activity slightly declined. This might be attributed to the greater toxicity of H_2_O_2_ under salt stress, where CAT and POD play complementary roles in its removal. In this case, CAT served as the primary enzyme for H_2_O_2_ decomposition in maize (Zrig et al., 2020). Meanwhile, plant growth status also improved significantly with increased organic fertilizer input.

In summary, salinity in mildly saline soils had limited adverse effects on maize. The combined application of organic fertilizer effectively prevented excessive vegetative growth and activated the antioxidant defense system, thereby enhancing photosynthetic efficiency and ultimately increasing yield, with the 50% organic nitrogen treatment showing the best performance. In moderately saline soils, maize was more sensitive to salt stress, which negatively affected its antioxidant system, growth, and photosynthesis. However, organic fertilizer addition enhanced stress resistance by stimulating antioxidant enzyme responses. Based on improvements in growth traits and photosynthetic performance, the 100% organic nitrogen treatment resulted in the highest yield under moderate salinity.O_2_^−^

## 5 Conclusion

To illustrate how the combined application of organic and inorganic nitrogen affects the photosynthesis and antioxidant properties of maize in soils with different degrees of salinization, this study selected maize fields with mild and moderate salinization. It set up field experiments with different organic-inorganic nitrogen application ratios. The main conclusions are as follows:

1. Under moderate salt conditions, growth indicators (such as plant height, leaf area index, biomass, chlorophyll content, and yield) and photosynthetic characteristics (including net photosynthetic rate, transpiration rate, stomatal conductance, and intercellular CO_2_ concentration) were significantly lower than those under mild salt conditions. In contrast, MDA content and antioxidant enzyme activities (superoxide dismutase (SOD), catalase (CAT), and peroxidase (POD) were significantly higher.
2. Mild salinity exerted limited effects on maize growth. The application of organic nitrogen prevented excessive vegetative growth, improved effective photosynthetic area and photosynthetic traits, and enhanced SOD activity. A 50% substitution of inorganic nitrogen with organic nitrogen resulted in relatively high yields. Under moderate salinity, increasing the proportion of organic nitrogen generally promoted maize growth traits, photosynthetic performance, and CAT activity, with full substitution (100%) of inorganic nitrogen by organic nitrogen yielding the highest grain production.
3. Correlation analysis revealed that in mildly saline soils, maize yield was significantly and positively correlated with photosynthetic parameters and SOD activity (P< 0.01), while in moderately saline soils, yield was significantly and positively correlated with photosynthetic parameters and CAT activity (P< 0.01). Structural equation modeling further indicated that in mildly saline soils, nitrogen application improved yield primarily through enhanced photosynthetic performance, whereas in moderately saline soils, yield formation was driven by an integrated influence of growth traits, photosynthetic parameters, and CAT activity.

This study clarifies the yield improvement mechanism of maize under saline conditions from the perspective of photosynthesis and antioxidant responses influenced by OIN application, providing a theoretical basis for saline farmland management. Future research should further explore the mechanisms of OIN effects under higher salinity levels to optimize nutrient management strategies for salt-affected soils.

## Data Availability

The original contributions presented in the study are included in the article/supplementary material. Further inquiries can be directed to the corresponding authors.
